# Evaluating the effectiveness of a multifaceted, multilevel continuous quality improvement program in primary health care: developing a realist theory of change

**DOI:** 10.1186/1748-5908-8-119

**Published:** 2013-10-08

**Authors:** Gill Schierhout, Jennifer Hains, Damin Si, Catherine Kennedy, Rhonda Cox, Ru Kwedza, Lynette O’Donoghue, Marea Fittock, Jenny Brands, Katherine Lonergan, Michelle Dowden, Ross Bailie

**Affiliations:** 1 Menzies School of Health Research, Level 1, 147 Wharf Street, Spring Hill, Queensland, Australia; 2 Queensland Department of Health, Level 6, William McCormack Place, 5 Sheridan Street, Cairns, Queensland, Australia; 3 Maari Ma Health Aboriginal Corporation, 443 Argent Street, Broken Hill, New South Wales, Australia; 4 Curtin University of Technology, Kent Street, Bentley, Western Australia, Australia; 5 Menzies School of Health Research, Building 58, Royal Darwin Hospital Campus, Rocklands Drive, Tiwi, Northern Territory, Australia; 6 Ngalkanbuy Health Service, Gakuda Street, Galiwinku, Northern Territory, Australia

**Keywords:** Quality improvement, Evaluation science, Realist evaluation, Primary health care, Program theory

## Abstract

**Background:**

Variation in effectiveness of continuous quality improvement (CQI) interventions between services is commonly reported, but with little explanation of how contextual and other factors may interact to produce this variation. Therefore, there is scant information available on which policy makers can draw to inform effective implementation in different settings. In this paper, we explore how patterns of change in delivery of services may have been achieved in a diverse range of health centers participating in a wide-scale program to achieve improvements in quality of care for Indigenous Australians.

**Methods:**

We elicited key informants’ interpretations of factors explaining patterns of change in delivery of guideline-scheduled services over three or more years of a wide-scale CQI project, and inductively analyzed these interpretations to propose fine-grained realist hypotheses about what works for whom and in what circumstances. Data were derived from annual clinical audits from 36 health centers operating in diverse settings, quarterly project monitoring reports, and workshops with 12 key informants who had key roles in project implementation. We abstracted potential context-mechanism-outcome configurations from the data, and based on these, identified potential program-strengthening strategies.

**Results:**

Several context-specific, mechanism-based explanations for effectiveness of this CQI project were identified. These were collective valuing of clinical data for improvement purposes; collective efficacy; and organizational change towards a population health orientation. Health centers with strong central management of CQI, and those in which CQI efforts were more dependent on local health center initiative and were adapted to resonate with local priorities were both favorable contexts for collective valuing of clinical data. Where health centers had prior positive experiences of collaboration, effects appeared to be achieved at least partly through the mechanism of collective efficacy. Strong community linkages, staff ability to identify with patients, and staff having the skills and support to take broad ranging action, were favorable contexts for the mechanism of increased population health orientation.

**Conclusions:**

Our study provides evidence to support strategies for program strengthening described in the literature, and extends the understanding of mechanisms through which strategies may be effective in achieving particular outcomes in different contexts.

## Background

Over the past ten years the Audit and Best Practice for Chronic Disease Project (the ABCD project) has developed and supported quality improvement tools and processes in primary health care centers across Australia, with a focus on centers that serve predominantly Indigenous populations. We have previously reported on enablers and barriers to participation in the project by health centers, with the objective of informing the development of more effective strategies for supporting uptake [1]. In this paper, our focus is on describing how contextual and other factors may interact to influence service delivery outcomes, particularly the desired outcome shared by most continuous quality improvement (CQI) initiatives, that of improved delivery of recommended care processes.

Variation in the effectiveness of CQI interventions has been commonly reported [2], and a range of theories, models, and empirical studies have advanced the understanding of this variation. However, there is a lack of understanding about how the various drivers of CQI effectiveness that have been identified in the health context interact with one another and with contextual factors to achieve desired outcomes [3]. There are few published empirical studies in this area from primary health care settings, and little clear guidance to policy makers or planners regarding how and in which circumstances CQI interventions could be modified to strengthen desired impacts in primary health care [3,4]. Our study makes an effort to address this gap.

The ABCD Project was designed to support best practice in prevention and management of chronic disease in Indigenous primary health care services in Australia. There are many different CQI implementation models described in the literature, with the ABCD CQI project sharing characteristics with what has been termed ‘integrated CQI’, that is, CQI models that are multi-site and multi-faceted and aim to achieve change at various levels of the system [5,6]. Between 2002 and 2006, the project used participatory action research methods at the organizational level to introduce and examine the impact of CQI in 12 remote Indigenous primary health care centers [7-9]. The extension phase of the project (2005 to 2009) was designed to examine the operational and policy requirements of expanding the ABCD model to other geographic locations, and to other core components of primary health care, including maternal and child health and mental health [10]. The project design drew on diffusion of innovation theory [11] (the ‘how’) and on CQI theory and methods (the ‘what’). By the end of 2009, the tools and processes developed through the project had been used in about 130 Indigenous primary health care centers across the country, including 69 services which were formally enrolled in the project research activities. Ethics approval was obtained from research ethics committees in each jurisdiction.

At the health center level, health centers were supported to conduct annual quality improvement cycles (plan-do-study-act) and a web-based information system provided participants with real-time analysis of their performance data, and capacity to compare it with others in their region and across the project. Health center staff were supported through their annual CQI cycles by state/territory co-ordinators (referred to as ‘hub co-ordinators’) appointed for this purpose. At the regional level, hub co-ordinators based in each of the five regions where the project was operating, had links to regional level management and academic support. In some regions, health boards, or umbrella organizations managing a group of health centers, took on implementation co-ordination roles, including sharing of lessons between organizations within their group. At the national level, the project core, based in an academic institution, undertook ongoing refinements to project design to ensure consistency with national and international evidence-based guidelines, and hosted annual feedback and planning meetings to which all participating health centers were invited. A number of project staff and affiliated researchers were engaged with the development of national and jurisdictional chronic disease policy [12]. Previous project publications have described how the diversity of health centers participating in the ABCD project took up the project in different ways [1], and the considerable variation in their delivery of services that are scheduled at specific intervals according to best practice guidelines (‘guideline-scheduled services’) [7-9].

In this paper, we combine inductive analytic approaches with principles of realist evaluation [13] to identify fine-grained hypotheses about how, and in what contexts, the ABCD CQI project may have achieved changes in delivery of guideline-scheduled services, focusing on services for diabetes and preventive care. The overall aim of the study is to identify how, why, and in what contexts CQI in primary health care may achieve various outcomes—and to share findings of relevance to implementation researchers, managers, and planners.

## Methods

### Research strategy

We elicited the interpretations of key informants regarding the factors explaining patterns of change in delivery of guideline-scheduled services during implementation of a wide-scale CQI project, and analyzed these interpretations in order to propose fine-grained realist hypotheses about what works for whom and in what circumstances. Theoretical explanations of this kind, or ‘middle-range theories’ ‘…involve abstraction… but [are] close enough to observed data to be incorporated in propositions that permit empirical testing’ [14].

Realist evaluation starts with program theory, uses theory to guide data collection and analysis, and ends with refined program theory. The program theory in realist evaluation specifies a relationship between context, mechanism, and outcome—or ‘CMO’ configuration (CMO)’ [13]. A key methodological challenge in applying realist evaluation in health systems research is identifying middle-range theory [13,15]. Our research strategy of developing middle-range theory based on program implementers’ views is consistent with recommendations from a recent overview of the use of realist evaluation methods in health systems research, which proposes this as an explicit additional step in situations where there is little to inform a-priori development of an appropriate middle-range theory [15]. The specific research objective of the study was to empirically identify CMO configurations or ‘middle range theory’ that may explain the effectiveness of CQI at health center level.

### Data sources and analysis

**Figure 1:**
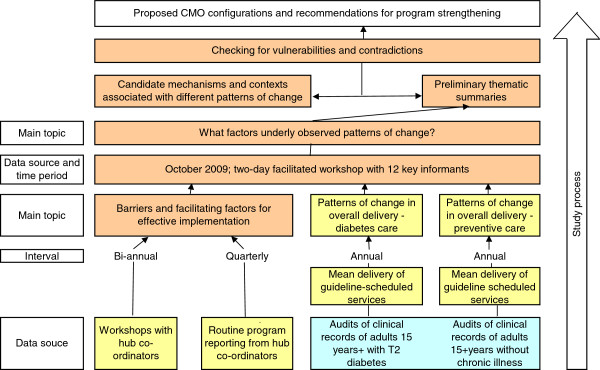
Outline of data sources and study process.

For the purposes of developing CMO configurations, or middle-range theory, we defined outcomes at the micro-system (health center level) as the starting point of the enquiry. There were 36 health centers across the States/Territories that had participated in the ABCD CQI project for three or more full annual QI cycles. As the analysis presented in this paper is concerned with achievement of improvements in quality of care over time, the study focused on these 36 health centers. These included both large and small health centers, in remote, regional, and urban locations. The data sources that were drawn on, and the approach followed is presented diagrammatically in Figure 1, and outlined below.

Hub co-ordinators responsible for supporting health centers with implementation, provided regular quarterly reports to the project management committee on implementation progress (16 to 18 reports over four to five years for each hub coordinator; ‘Routine program reporting’ in Figure 1), and participated in a series of workshops over the course of the project (eight workshops over four years; ‘Workshops with hub-coordinators’ in Figure 1). In these workshops, the hub coordinators, the project manager, and key investigators worked to define the salient factors for effective implementation of the ABCD CQI project based on their experience, and in relation to factors identified in the literature. As a result of their experience on this project and their more general experience of working with services involved in the project, many of the workshop participants had insight into the day-to-day operations of many of the participating health centers. Through the workshop process we were thus able to compare and contrast the insights of various workshop participants into factors that were present across different health centers that showed similar patterns in their data. The analysis was therefore not dependent only on the insights of one individual into the circumstances of each health center. The workshop process was complemented by ongoing liaison between the hub co-ordinators, key investigators, and the project manager in iterative cycles of clarification of concepts and refinement of the analysis.

### Selection and presentation of outcome patterns

**Figure 2:**
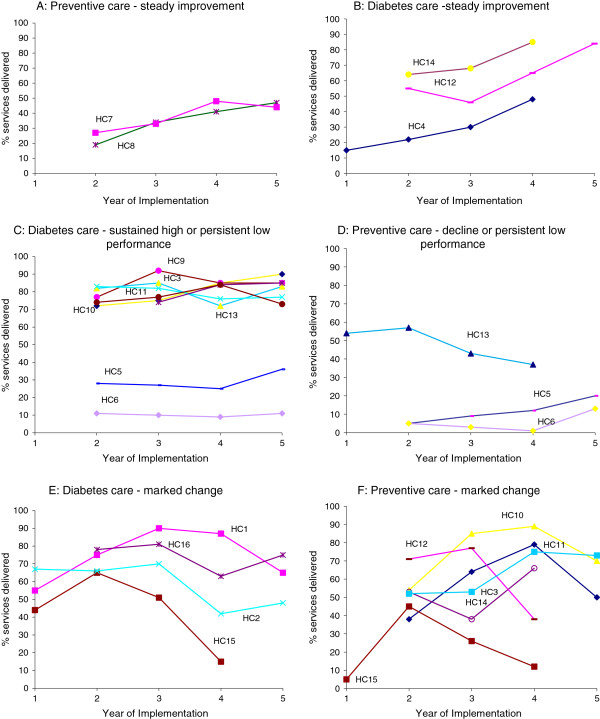
**Illustrative health center patterns of change for diabetes and preventive care.** HC: health center; panels **A-F** illustrate the different patterns of change.

Drawing on clinical audit data collected by each health center as part of their CQI cycles, we constructed a measure of overall delivery of scheduled services for diabetes and for preventive care to well adults for each health center for each year of participation. Quantitative methods to examine variation on these measures across the project have been previously reported [7,8]. From the diverse range of patterns of change in the clinical audit data for each health center over the duration of the study, we identified a number of major qualitative patterns of change (along the lines of the data presented in Figure 2). We did this separately for patterns of change in delivery of diabetes-related services and for delivery of preventive services (‘Patterns of change in overall delivery of care for diabetes/preventive care’ in Figure 2). It was inevitable that some health centers did not show patterns as clearly as others, and that for some health centers, respondents were less able to identify explanation for patterns of change. The selection of trend lines shown in Figure 2 are for health centers that showed trends in delivery of care that were most clearly illustrative of the patterns that were discussed, and those for which we had the best explanatory data. Further details on how these measures were constructed and patterns of change identified, are provided in Additional file 1.

### Key informants perspectives on factors influencing outcome patterns

Late in the final year of the extension phase of the ABCD project, graphs showing changes over time in the audit data for each health center, categorized by the major qualitative patterns of change, were presented and discussed in a workshop with the regional hub-co-ordinators, key investigators and other members of the project management committee (‘two day facilitated workshop’ in Figure 1). These 12 key informants had detailed insight into the experience of engagement by health centers in the CQI process through their involvement in supporting local and regional level implementation, and had access to quarterly progress reports for the three years of project implementation. During the workshop discussions, informants were asked to consider the situation of each health center over the time covered by the study, and give their perceptions on what contributed to the patterns of change in delivery of services. Discussions were recorded and transcribed verbatim so that the raw data could be systematically analyzed.

### Analysis of the key informants’ perspectives

As a first step a preliminary thematic summary of key informants’ interpretation of factors explaining outcome patterns was produced (‘Preliminary thematic analysis’ in Figure 1). This thematic summary was based on project implementers’ understanding of what could explain the various outcome patterns observed in the quantitative data. Members of the study team met to discuss the emerging themes, and the thematic summary was circulated and refined on the basis of feedback and discussion using a group consensus approach to strengthen the validity of our findings. Broad preliminary themes that were identified included: data and data systems; regional support; leadership and management; organizational culture; approach to service delivery; community linkages; adequacy and stability of staffing; and ownership and management of CQI. Following this, the themes were refined and categorized into ‘mechanism’, ‘context’ or ‘intermediate outcome’, on the basis of the realist conception of mechanism and outcomes [13], and drawing on the typology of mechanisms in the context of program evaluation outlined by Astbury and Leuuw [14] (‘candidate mechanisms and contexts’ in Figure 1). In identifying mechanisms and the contexts that enabled them, we drew on organizational theories of change [16] and theories and models of quality improvement [17]. We drew only on aspects of these theories and models that could be identified or grounded in our qualitative data and were consistent with program logic and activities.

Through an iterative process of checking data against candidate CMO propositions, we sought to identify and refine key mechanisms that appeared to best explain health center level changes in delivery of guideline scheduled services over time (‘outcomes’) and what contexts seemed to enable or inhibit these mechanisms (‘checking for vulnerabilities and contradictions’ in Figure 1). The initial CMO configurations were redefined and refined through a process of looking across the different patterns of change, at ‘positive’ and ‘negative’ cases, and where the same mechanism appeared to be enabled by different contexts. We attempted as far as possible to include all of the common factors that were identified as explaining each of the outcome patterns. Based on transparent reasoning, we identified specific strategies for strengthening wide-scale CQI programs in identified contexts, which may merit further testing and refinement.

## Results and discussion

Data presented in Figure 2 demonstrate wide variation in baseline level of performance and divergent patterns of change achieved by health centers for overall diabetes and preventive care. As will be evident in the presentation of results below, the participating health centers also showed considerable heterogeneity in their contexts, activities, and outcomes.

We identified three main mechanisms, and seven potential CMO configurations as candidates to explain different patterns of change. Table 1 shows these CMO configurations, together with exemplar quotes, and a summary of the specific inputs of the ABCD CQI project relevant to each of the proposed mechanisms. In the text below, we describe each of the mechanisms; what contexts appeared to trigger or inhibit each mechanism; and what outcome patterns resulted. Specific health center examples are presented in order to illustrate our reasoning. The numbering of the trend lines shown in Figure 2 allows linking of the textual explanation of factors underlying various patterns of change to the trend lines for specific health centers. In the presentation of results below, where contexts are discussed they are numbered in order of citation and referred to as C1, C2.... A similar approach was followed in discussion of mechanisms (M1, M2…). The CMO configurations are reframed into proposition statements, encapsulating the most important findings of use to researchers, managers, and practitioners (see section "List of CMO configurations reframed as proposition statements"). Potential strategies for strengthening wide scale CQI projects in different primary health care contexts and the lines of reasoning that led to them are provided in Table 2.

**Table 1:** Proposed CMO configurations explaining how a wide-scale CQI model in primary health influences care delivery

**Summary of salient ABCD CQI project inputs**	**Potential contexts**	**Plausible mechanisms**	**Potential outcomes**	**Exemplar quotes**
● Health center staff participate in annual predominantly paper-based audit processes, interpretation of reports and systems assessment and action planning that use data derived from clinical audit, as a starting point for change	Centralized management style; regional board committed and involved in CQI implementation (C1)	Collective or shared valuing of clinical data for improvement purposes (M1)	● Temporary declines and instability as services get used to new systems. Major revision of clinical record keeping; centralized ‘cleaning up’ process to standardize reporting across health centers.	‘Across our region we did a concerted effort for documentation for diabetes services…and so certainly the improvement [in the early years] would just have been about documentation, so having somewhere to write things…I think all was about documentation. But 2006 to 2007 I think there was a concerted effort. The chronic disease strategy really kicked in and that was when, at some point during that period, [name] had her lights on moment. [we understood] the focus of how important doing the right processes at the right time was’.
			● Marked changes (HC1 & 2 Figure 2E).	
● Automatic generation of reports from clinical audit through a web-based information system.	Local ownership of CQI (devolved management style); competent staff in management roles; managers and clinicians with an interest in chronic disease and in clinical and population health data (C2)	Collective or shared valuing of clinical data for improvement purposes (M1)	● Use of non-core strategies such as follow up of individuals receiving poor care identified in clinical audit, used to highlight clinical relevance of data (HC 7 & 8 Figure 2A).	‘Doctor [name] was always really, really interested in the data…where he saw really big increases in ACRs and that, he would want to know who were the people that were being audited in terms of following those up. So he was very good with that. And then of course [name] is their quality improvement person… they were standardising their filing system right across that region, which she led, and so [the data] were quite easily accessible’
● Ability for health centers to adjust reporting (format, indicators etc.) to suit local reporting requirements and accountabilities			● Sustained high performance or marked change to improvement (HC 3 Figure 2C &2F).	
● Engagement of champions and change agents at different levels of the health system to promote uptake of the project	Poor management, uncertainty and confusion over role definitions. (C3)	Collective or shared valuing of clinical data for improvement purposes (M1)	● Limited changes in data systems; frustration and confusion about ongoing involvement in CQI.	‘A lot of health workers. Been there for a long time, and I asked them what, sort of asked what their training was. Why aren’t they doing like blood pressures and blood sugars…They said they were not allowed. They’ve been told by management they’re not allowed.…that was part of their training though that, you know, I’m a health worker and that’s part of my training. But yeah, a lot of them have been there for 15 years. They just didn’t have a focus. We actually wondered what their existence was about’.
● Ongoing refinement of the project to maximize synergies with major policy initiatives			● Poor performance or declines in care (HC5 & 6 Figure 2C and 2D).	
● Processes and tools that brought together different health care professionals and managers to share ideas for service performance and improvement activities	Regional or organizational infrastructure supportive of networking for CQI and centralization of some tasks. Positive prior history of collaboration (C4)	Collective change efficacy (M2)	● Appropriate reflection on salient comparison group; formation of networked communities	‘Have good communication systems… share ideas between the different health centers. And a strong focus on education through regional support teams… use video conferencing as well as regular visits.. and its very vibrant, like they are always out there’.
			● Sustained high performance or marked change to improvement (HC3 Figure 2F and 2C).	
● Annual planning meetings, meetings, teleconferences and sharing of experiences between health centers	Organizational culture unsupportive of collaboration. Health centers see themselves as being in competition (C5)	Collective change efficacy (M2)	● Inappropriate reflection on performance and early fatigue	‘Cause, yeah, when I first started they were really eager, you know, like doctors were all eager to see how, cause there’s three clinics in [name of city]. They were all competing with each other, who’s going to be the best, and who’s going to give the best service, so but it’s just worn off’.
● Provision of benchmarking data, allowing health centers to reflect on their performance in relation to that of others			● Persistent low performance or declines in care (HC5 and HC 6, Figure 2C and 2D).	
● Application of CQI to a wide range of health outcomes and service populations (diabetes, preventive health, maternal health, child health), and a range of care processes	Pre-existing favorable context of patient and community oriented care, supported by stable effective outreach workers and good regional co-ordination for CQI (C6)	Organizational change to encompass a population health orientation (M3)	● Recognition of value and roles of Aboriginal Health Workers in outreach and linking this to service delivery.	‘With [NAME] they had the self management program there, and they get a lot of stuff outside the health center.. it was about promoting good health in the community, working with the store [for supply of healthy food in this remote community], and those places. A lot of health promotion activities were going on with those health workers there. .. Population lists were being improved and a better understanding [in the context of transient populations and population movement]’.
			● Developing greater consistency in provision of general practitioner services.	
● Processes that brought different service delivery professionals together to reflect on health center performance (for example, outreach workers and clinic-based staff)			● High performance and marked change to improvement (HC10 Figure 2C and 2F).	
● Regionally based co-ordinator positions supported population health planning and multidisciplinary team approaches to chronic disease care	Staff who can identify with patients and have the skills to take broad ranging action, including clinical action and action related to data system development and use, coupled by regional support and co-ordination (C7)	Organizational change to encompass a population health orientation (M3)	● Priority-driven resource allocation decisions.	‘P1: Well [NAME] is passionate about making sure all the diabetics [are well cared for] …P2: He was also a diabetic wasn’t he? P1: Yeah. He had a personal drive and he was cardiac nurse, so any cardiac stuff that was related to diabetes, you know, he could tell people when they were being sent to Adelaide and you know, he did all that sort of advice as well…And what he did though was set up the big clean up of the data system. And started extracting reports and cleaning up the population base’.
			● Mixed patterns (high performance or marked change to improvement in diabetes and low in prevention or vice versa) (HC12 Figure 2B and 2F and HC13 Figure 2C and 2D).	

**Table 2:** Potential strategies for strengthening wide-scale CQI projects to enhance clinical performance in different contexts

**Context**	**Proposed mechanisms and reasoning for recommended strategies**
Mechanism 1: Collective or shared valuing of clinical data for improvement purposes: if health centers expect their clinical audit data to be fit for the purpose of QI, then they will be more motivated to use these data for service improvement as envisaged by the CQI model.	
Centralized management style; regional board committed and involved in CQI implementation (C1)	● If centralized management of CQI institutes revision of clinical record keeping systems, participating health centers will develop collective or shared valuing of clinical data for improvement purposes, and will in fact use the data for performance improvement, resulting in improvements in care delivery.
	● If this works because of the expectations of the potential for data to support CQI (for example, through social mechanisms such as the ‘self fulfilling prophecy’), then
	wide-scale CQI projects could encourage health centers sharing this context to enter CQI processes with optimism, and use processes as a way to motivate for improvements in clinical record keeping even where good quality data on care processes are not consistently available at the outset
Local ownership of CQI; competent managers with interest in chronic disease and clinical and population health data (C2)	● If clinical staff use data in non-core ways to illustrate the applicability of data and importance of record keeping, health centers participating in these initiatives will develop collective or shared valuing of clinical data for improvement purposes, and will in fact use the data for performance improvement, resulting in improvements in care delivery.
	● If this works because of the adaptive potential of the project then
	wide-scale CQI projects could develop examples of different presentation formats of audit data, and of CQI processes to illustrate adaptive potential more strongly, demonstrating their capacity to resonate with different organizational cultures and vision
Poor management, uncertainty and confusion over role definitions (C3)	● If poor overall management and role confusion detracts from health center staff perceptions of the value of their data, health centers participating in wide scale CQI projects are less likely to develop shared valuing of clinical data for improvement, and will be less likely to use the data for performance improvement, constraining the potential for improvements in care delivery, and discouragement (negative feedback loop).
	● If this context is a key constraint on the effectiveness of CQI, then
	interventions targeting unfavorable organizational contexts should be developed, prior to, or in parallel with, CQI implementation
Mechanism 2: Collective efficacy - If health center staff have a strong sense of shared belief of achieving improvement through the CQI project, then they will be more motivated to attempt changes to improve service delivery as envisaged by CQI, devote considerable effort to it, and persist in the face of difficulties.	
Infrastructure supportive of CQI networking; positive prior history of collaboration (C4)	● If regional/organizational infrastructure is supportive of networking for CQI, and networks are formed, health centers will attempt changes, put effort into changes and show persistence, resulting in improvements in care delivery.
	● If this works because of informal social control enacted under conditions of social trust, then
	wide-scale CQI projects could encourage greater density of networks between health centers in this context, transparent sharing of information and experiences related to CQI
Organizational culture unsupportive of collaboration (C5)	● If organizational culture is unsupportive of collaboration, inappropriate competitiveness and early fatigue and disillusionment will result. If this ‘works’ because of lack of co-operation with social control, related to lack of social trust, then
	wide scale CQI projects could seek to identify health centers sharing this context, and aim to build sufficient trust for collaborative networking to take place
Mechanism 3: Organizational changes towards a population health orientation - If health centers share an understanding of their role as supporting health of their service and community populations, not just those presenting for care, then they will engage in activities outside of the health center, build trust with community members, instituting changes for service improvement that are consistent with community needs, and therefore more likely to be acceptable to the community and lead to greater demand for services, and increased delivery of guideline scheduled services – as long as the guidelines and indicators measured are consistent with community needs.	
Pre-existing favorable context of patient and community oriented care, supported by stable effective outreach workers and good regional co-ordination for CQI (C6)	● If organizational culture has a strong external focus, participation in CQI may enable clearer understanding of unmet need/under delivery, helping health centers to galvanize to improve care, and will use these data for performance improvement.
	● If this works because of the role of CQI in providing information on population health needs, then
	wide-scale CQI projects could be designed as broad integrated models as these will be more likely to trigger change towards a population health orientation than narrow CQI models that focus on a more limited range of clinical targets
Staff who can identify with patients and have the skills to take broad ranging action (C7)	● If key individuals are motivated and empowered to take broad ranging action, and have the support to do so, then they will actively participate in wide-scale CQI projects, and use these as a tool to initiate improved care delivery
	● If this works because of the role of individual level enthusiasm in promoting change, then
	wide-scale CQI projects could seek to proactively build the skills and development of enthusiastic clinical leaders in promoting overall performance improvement across the scope of clinical care

### List of CMO configurations reframed as proposition statements encapsulating the most important key findings

1. The mechanism of collective or shared valuing of clinical data for improvement purposes (M1) can be enabled by the context either of centralized management style of a regional board, where CQI and change is centrally-led (C1), or in situations where implementation of CQI is devolved to local health center level, providing that local health centers have competent and experienced staff with capacity to appreciate potential of data to improve clinical care (C2). Staff capacity and co-operation are likely to be key constraints on this mechanism, particularly in situations of relative isolation of services and small staff complements (C3). Further, this mechanism may also be enabled at the macro level. Where this occurs, its influence at a health center level may be more important in the context of local devolution of CQI implementation (C2) compared to centrally-led implementation (C1).

2. Collective change efficacy as a mechanism (M2) can be enabled by the existence of regional or organizational infrastructure that supports networking for CQI, particularly where there is positive prior history of collaboration (C4). Remote and geographic dispersion of health centers may be an additional favorable contextual factor for change efficacy as networking type activities between health centers may be more valued in situations of relative isolation. Competitiveness and organizational culture unsupportive of collaboration where there is role confusion and/or poor co-ordination between service providers (C5) is likely to inhibit activation of this mechanism.

3. Organizational change to encompass a population health orientation (M3) can be enabled by the context of stable effective outreach staff and good regional co-ordination (C6), and by the contexts in which health center staff identify with patients, and have leadership skills to take broad ranging action in CQI (C7). Resource constraints and financial incentive structures may act as moderating influences on the outcome patterns achieved.

### Mechanism 1: Collective valuing of clinical data for performance improvement

Our analysis suggested that one of the processes through which improvement came about was through a collective or shared valuing of clinical data for improvement purposes. This was expressed through the attitudes of health service staff and managers towards clinical data. For example, in explaining marked change to improvement, and sustained high performance, informants spoke about individual health center staff who were passionate and committed to using clinical data to improve service delivery, along with organizational initiatives to develop and improve the capability of clinical information systems to provide data for this purpose (Table 1, exemplar quotes).

Our analysis identified three main contexts that appeared to trigger (or inhibit) this mechanism (M1). These are outlined below.

### C1: Centralized management style; regional board committed and involved in CQI implementation

One of the challenges faced by health centers implementing the ABCD CQI project was the inconsistent state of development of clinical record keeping systems, and the constraints this imposed on their ability to collate adequate data on care processes through clinical audit [1]. Some health centers undertook major revisions of their clinical record keeping systems in response to the need highlighted by the project. Although improvements in clinical record keeping occurred in a number of different regions on a limited scale, revision of record keeping systems was most marked in the one region where CQI processes were supported by strong central management and regional support systems (C1). The board in this particular region, responsible for a cluster of health centers, implemented a wide ranging data quality and service improvement process, including improving clinical information systems and documentation of service delivery, record filing, developing accurate service population lists and disease registers, and implementation of recall and reminder systems. For health centers in this region that showed improvements in service delivery, key informants identified the attention paid by the regional board to improving data systems as an important explanatory factor for these patterns of change (for example, HC12 and HC14 in Figure 2B). Improved record keeping appeared to be particularly relevant in early years of implementation. In later years, patterns of change to improvement were explained by capacity and motivation of health center staff.

Several health centers within the same region showed marked changes in delivery of services, particularly related to diabetes care, as practitioners were learning how to use new data systems (Figure 2E, HC1 and HC2). Broad ranging efforts to improve documentation and data systems sometimes resulted in patterns of improvement (O1) and sometimes they contributed to short-or medium-term inconsistencies in observed patterns of change as clinical record keeping systems were being modified and updated (O2).

For two of the health centers that shared this context, but that showed declines in service delivery after initial improvements (O3), staffing constraints appeared to be key contextual influences on their ability to sustain higher performance. In one of these health centers, a senior nurse who played a major role in service delivery, became disgruntled because of perceived lack of support, and refused to work with regional systems supporting best practice across the scope of clinical care. This refusal coincided with loss of a service provided by a visiting endocrinologist—this specialist provider had provided services to diabetes clients, and withdrew this service provision during the period covered by the year three audits. The marked change to decline for this health center was explained by the combined effect of these two constraints (HC15, Figure 2E and 2F). In another health center (HC2), the combined effect of implementing the new data system, together with decline in staff capacity as a result of a high performing Aboriginal health worker taking on part-time study (with consequent less involvement in service delivery), were identified as explaining the marked decline in performance between years three and four.

The findings presented above led to the hypothesis that one of the ways in which this CQI project achieved its effects, was through collective valuing of clinical data for improvement purposes, enabled by strong central management of CQI. We noted the role of the social situation or event (in this case the regional board initiating wide-scale improvements in data systems) in shaping the response of individual health centers. Applying the reasoning of the self-fulfilling prophecy [18], we hypothesize that collective or shared valuing of clinical data for improvement purposes may have ‘worked’ to achieve desired outcomes at least partly because of the principle that expectations are brought about because of the belief that they are justified. Although there may be rival explanations, this underlying social mechanism was a plausible fit with the patterns in our data, and suggests that collective valuing of data may be strengthened in similar contexts, through strategies designed to maintain confidence and optimism in data (Table 2).

### C2: Local ownership of CQI (devolved management style); competent and supportive health center managers and clinicians with an interest in chronic disease and in clinical and population health data—either at health center or regional level

The presence of influential clinicians who were able to relate data to care improvement was commonly cited as a factor underlying sustained high performance or marked change to improvement. For example, for two of the health centers showing these patterns, key informants noted that clinicians had been enthusiastic to identify and follow up individuals for whom the clinical record audit showed gaps in care processes. While the follow up of individuals is not a core part of CQI activities, these particular clinicians had used this process as a means to highlight the value of data and to motivate for better clinical record keeping. The critical aspect here seemed to be the sense of urgency and motivation to make use of clinical records to improve patient care (Table 1).

Where there was limited capacity at local level, leadership in relation to data orientation could be supplemented to a certain extent at regional level. For example, for two health centers located in remote communities that showed steady improvement (HC7 and HC8, Figure 2A), key informants identified competent and experienced staff in regional management roles, managers with an interest in chronic disease and in clinical and population health data as contributing to these patterns of change. The jurisdiction in which these two health centers (HC7 and HC8) were located, introduced an adult health check template during the period of implementation of the ABCD CQI project. This jurisdiction-wide strategy to improve delivery of preventive services was noted as one of the factors contributing to improvements in preventive care in these particular services. Both services also received funding from an Australian government initiative that provided some support for quality improvement activities, including networking and coordination between services. The ABCD CQI project enabled health services to generate data for reporting for this government initiative, and the congruence between the project and this and other national and State/Territory initiatives appeared to be an important characteristic of the project in relation to developing shared valuing of clinical data for improvement purposes.

The influence of macro level contexts appeared particularly important as an explanation for change where CQI implementation was based primarily on local level initiative (C1), rather than being supported through regional systems.

### C3: Poor management, uncertainty and confusion over role definitions, sometimes including lack of a clear and consistent definition of service populations

A key characteristic of the two health centers that showed persistent low performance for both diabetes and preventive care (HC5 and HC6, Figure 2C and 2D) was that they showed little interest in using clinical data for CQI. In explaining the low performance of these health centers, key informants noted that these health centers were managed by a central organization that had experienced high turnover in the CEO position, had limited management commitment to CQI, and consequent delays and interruptions to CQI processes. These health centers were staffed by nurses and health workers, did not offer any on-site medical care/general practitioner (GP) services, and serviced transient and mobile populations. Several other health centers that shared these additional characteristics, but were better managed and had greater commitment to using data for CQI, had achieved significant improvement in delivery of preventive services (though not diabetes services) through establishing partnerships with local GPs.

### Mechanism 2: Collective change efficacy

Collective change efficacy relates to the belief that one’s organization can achieve the desired change in the specific setting [19]. Collective change efficacy was expressed through program implementer explanations for outcome patterns that highlighted the role of networks, effective teamwork, and the role of intra- and inter-organizational learning in supporting performance improvement.

### C4: Regional or organizational infrastructure supportive of networking for CQI and centralization of some tasks and positive prior history of collaboration

In our analysis, regional networks and their communication systems were identified as a key factor in explaining patterns of change, and plausibly may have triggered the development of collective efficacy as a mechanism of performance improvement through CQI. For health centers demonstrating sustained high performance, steady improvement or marked change to improvement (HC11, Figure 2C and Figure 2F between years two and four; and HC3, Figure 2F), reference was made to specific communication systems used by the health board managing these centers, including regular cross-site visits and video conferencing. The health board managing this group of services encouraged the use of additional complementary strategies, such as a rapid plan-do-study-act cycle focusing on short-term care process targets in defined areas. Most of the health centers participating in such networks in our study were remotely located and geographically dispersed from one another and from other sources of support. These health centers had a positive prior history of collaboration that went beyond project inputs and activities. It seems plausible that remoteness and geographic dispersion of health centers may be an additional favorable contextual factor within this broad context, possibly owing to the added attractiveness of networking type activities in counteracting structural and operational isolation of remote services.

### C5: Organizational culture unsupportive of collaboration. Health centers see themselves as being in competition for its own sake

Where organizations were unsupportive of collaboration, and saw themselves as being in competition with one another, the mechanism of collective efficacy may have been inhibited. For the two health centers (both remotely located) that showed persistent low performance in both diabetes and preventive care (HC5 and HC 6, Figure 2C and 2D), inappropriate reflection on performance and early fatigue was identified as explaining this poor performance.

### Mechanism 3: Organizational change to encompass a population health orientation

This mechanism proposes that the ABCD CQI project may have achieved some of its effects through its ability to assist organizations to develop a population health orientation. Organizations with a population-health orientation are characterized by: provision of population based care, rather than care responsive only to those presenting for treatment; systems thinking; working across the care continuum from clinical prevention to palliative care and; and recognition that the system is primary care-led, with effective partnering with secondary and tertiary care [20]. Some health centers participating in the ABCD project shared many of the elements of population health oriented organizations, and this orientation was identified as a key context underlying ability of these health centers to sustain high levels of performance. The further development of population health orientation as a mechanism for performance improvement through CQI was enabled by strong regional co-ordination and support for CQI, coupled with stable effective outreach workers and health center staff who can identify with patients and have skills to take broad ranging action.

### C6: Stable effective outreach workers and good regional co-ordination for CQI

For some health centers demonstrating sustained high performance or steady improvements in diabetes and preventive care, their implementation of population-health outreach activities was cited as one of the factors underlying their ability to demonstrate improvements. For example, one health center with high performance in diabetes care, and marked change to improvement in preventive care (HC10, Figure 2C and 2F between years two and four) had developed a storyboard intervention to educate clients and community members about various issues of public health concern. In response to clinical audit data showing gaps in care processes for their service population, the health center staff extended and enhanced this storyboard initiative. For example, the health center developed a stronger proactive outreach approach to clinical preventive checks by including these as part of the work of Aboriginal Health Workers who used storyboards about smoking, health, nutrition, and hygiene done in association with clinical checks during outreach visits. This health center also made a concerted effort to access external support available for population health activities, including nutritional programs. Possibly owing to the presence of effective outreach workers and the support of a strong regional body, this health center was able to achieve these outcomes despite high turnover of clinical staff.

### C7: Staff who can identify with patients and have the skills to take broad ranging action, including clinical action and action related to data system development and use, coupled with regional support and co-ordination

The presence of specific staffing attributes was a favorable context for activation of the mechanism of organizational change towards a population health orientation.

For a cluster of health centers that showed improvement or high performance in diabetes care together with decline in preventive care (HC12, Figure 2B and 2F between years three and four; and HC13, Figure 2C and 2D between years two and four), informants noted that these health centers prioritized disease management ahead of preventive care for well adults. While health center priorities were partly influenced by resource constraints and financial incentives, they were also influenced by the priorities and competencies of individual staff members.

In explaining patterns of declining performance, frequent reference was made to key staff members who had left, and in some instances, not been replaced. In one of the examples already mentioned (HC12, Figure 2F), the marked change to decline in delivery of preventive services, was to some extent explained by loss of a key medical staff member and a senior Aboriginal Health Worker, concurrent with the relocation of the center to a new building. However since this health center was able to maintain, and improve on diabetes care during this same period (HC12, Figure 2B), this suggests that greater priority was afforded to diabetes care than to preventive care. It may also reflect the nature of the services that had been delivered by the staff member who left, and that responsibilities had not been effectively re-allocated. While there were a number of examples in which staffing constraints appeared to influence absolute levels of capacity to deliver guideline-scheduled services, staffing changes also appeared to influence patterns of change through changes in configurations and functioning of teams.

Health centers able to sustain performance in the face of staff turnover were characterized by strong regional support systems together with commitment to good health center systems (HC3, Figure 2F between years three and four; HC12 and HC14, Figure 2B). Conversely, where regional support was lacking, or regional function was not adequate, the ability of a health center to show improvement was compromised. In one health center (HC13, Figure 2C), a slight temporary decline in diabetes care in otherwise consistent high performance was seen in a health center that was affected by a regional shortage of medical staff in government managed centers, and a restriction of services to acute care only. A deliberate policy of not taking any blood samples for routine monitoring of chronic illness care was instituted because there was no medical follow up available for any abnormal results that might have been required. This regional crisis was associated with poorer performance in service delivery for this health center, but not for some of the others in the same region, because for this health center, the crisis coincided with a period of recurrent turnover of clinic managers. Performance in preventive care in this health center declined over the same period, and remained low, even when diabetes care returned to previously high levels after the severe shortage of medical staff was relieved.

### Linkages and inter-dependence of mechanisms

Overall, while we have identified three main mechanisms of change and discussed these separately, conceptually and practically these mechanisms are inter-linked. While our study was not designed to test any particular theory or conceptualization of organizational change, the mechanisms and relationships between them had some similarities with Weiner’s conceptualization of organizational change, which theorizes early implementation effectiveness [16]. In Weiner’s conceptualization, ‘change valence’, or how much organizational members value change as being worthwhile (similar in some respects to our ‘Mechanism 1’ – collective valuing of the use of data for improvement purposes), together with favorable informational assessment of task demands and resource availability, leads to collective efficacy (similar to our ‘Mechanism 2’). The inter-dependence of mechanisms identified in our study is also consistent with underlying social cognitive theory, which for example highlights reciprocal relationships between enactive mastery (*e.g.*, performance feedback) and collective efficacy, particularly where performance feedback is provided to the whole of the team.

## Conclusion

Using interview data about how key informants interpreted outcome patterns in health centers implementing a multi-faceted CQI project, our study, focusing on ‘middle-range theories’, aimed to articulate more precisely the potential causal linkages between program activities of the ABCD CQI project and outcomes that were achieved. To our knowledge, this is the first time mechanism-based explanation has been empirically developed from a large-scale CQI project in primary health care. While the insights from the paper may be of interest to leaders of smaller scale CQI projects, our key messages are for wide-scale projects and are particularly relevant to understanding ‘integrated CQI’ models in primary health care settings [5].

The ABCD CQI project inputs, and the mechanisms and contexts described are broadly similar to ‘necessary but not sufficient’ common conditions of effective QI reported previously [6]. Our analysis extends this and other broad understandings of the ‘pre-conditions for success’ to suggest how and why different conditions may achieve changes in delivery of care processes through both the design aspects of CQI (project inputs), and implementation context.

A particular strength of our study is its reflection on a key pillar of CQI—that of the use of data as a tool to achieve improvements in quality of care over a number of years of implementation, and how patterns of change are explained by those responsible for supporting CQI implementation. We recognize the possibility of confirmation bias as a threat to the validity of theory-informed data collection, and in considering its impact we note the wide range of literature that informed the evaluative fieldwork and the ongoing emphasis placed on reporting what was actually observed in practice; these aspects of the project would have minimized the potential effect of this type of bias. A related limitation of our study is that our data were derived from a relatively small number of key informants (n = 12) and, although these informants had detailed knowledge of the health centers included in the study and the process of project implementation, they were not necessarily all familiar with the range of effects operating at different levels of the health system. This may have influenced the nature of the data and the level of enquiry that was possible. For example, although the study findings suggested significant effects of social structures and environments in explaining outcome patterns, we were not able to identify the specific situational mechanisms that could explain how social structures and environments may have shaped improvement leadership and management commitment to CQI—or influenced other aspects of context or mechanisms.

Future directions for research suggested by this study include: further empirical inductive studies along the lines of the approach reported here but specifically designed to include a wider range of key informants and able to consider the whole chain of situation, action-formation, and transformational mechanisms [21] appropriate to multi-level multi-faceted CQI interventions; and more focused evaluation studies of CQI that include testing of fine-grained realist hypotheses.

The recommended strategies for modifying CQI projects for greater effectiveness in different contexts flowing from this study include those that should (if our hypotheses are correct) make contexts more favorable to implementation of CQI, or strengthen the action of the key mechanisms that we identified as explaining the patterns of change in our data. These strategies are consistent with previous literature. What our study adds, is that it identifies the context in which strategies should be applied, and this is based on a clear line of reasoning linking identified mechanisms, with program activities, context, and observed outcomes.

## Competing interests

One of the authors (RB) is the Scientific Director of One21seventy, a not-for-profit entity within Menzies School of Health Research that provides QI support on a fee for service basis to primary health care services across Australia. None of the authors receive financial support from One21seventy, and One21seventy is not providing any financial support for the preparation of this manuscript. The authors have no other competing interests in the preparation of this manuscript.

## Authors' contributions

RB conceptualized and led the overall project, and contributed to the development of the manuscript. GS played the lead role in the realist analysis and in preparation of the manuscript. All other authors were involved in data collection and interpretation. All authors read and approved the final manuscript.

## Supplementary Material

Additional file 1 Measures of service delivery and patterns of change. Click here for file
