# Supplementary material for: Evaluating the effectiveness of a multifaceted, multilevel continuous quality improvement program in primary health care: developing a realist theory of change
Source: Implement Sci. 2013 Oct 8;8:119. doi: 10.1186/1748-5908-8-119 (PMC4124892; doi:10.1186/1748-5908-8-119)
Supplement: Additional file 1 — Measures of service delivery and patterns of change. [file 1748-5908-8-119-S1.docx]

Additional File 1: Measures of service delivery and patterns of change

We constructed a measure of overall delivery of diabetes care (at the individual patient level) by examining delivery of the 13 service items which the clinical guidelines use across the states/territories recommended for people with diabetes. The sum of service items delivered was divided by 13 to create the overall measure (expressed as a percentage). The overall measure was averaged for each health centre (‘Mean delivery of guideline scheduled services’ in Fig 1). A similar process was followed for delivery of preventive services. This measure was constructed for each health centre for year of participation in the ABCD project.

We examined the patterns of change in delivery of services over time, and identified common patterns of change qualitatively. Some of the patterns identified were relatively self explanatory – for example, health centres showing ‘steady improvement’ or sustained high performance or sustained low performance could be fairly easily identified. Health centres showing ‘marked change’ (for example, health centres which showed an unexpected decline, after two relatively high performing years, or vice versa) were identified as a separate category, as it was felt that reasons underlying marked changes would be different from reasons underlying more steady improvements (or declines) in care. In some cases, changes in delivery of diabetes services and preventive services in a health centre mirrored one another (e.g. a pattern of steady improvement or sustained high performance in diabetes care was matched by a pattern of steady improvements or relatively high performance in preventive care), but this was not always the case.

These differences in patterns of change were identified as potential fruitful areas of enquiry in developing an understanding of the underlying mechanisms of change and the contexts that trigger or inhibit activation of these mechanisms for different aspects of primary health care quality improvement.
